# Association of Metabolic Syndrome Components and Overactive Bladder in Women

**DOI:** 10.7759/cureus.14765

**Published:** 2021-04-30

**Authors:** Corc Baytaroglu, Emrah Sevgili

**Affiliations:** 1 Cardiology, Avcılar Hospital, Istanbul, TUR

**Keywords:** bmi, glucose, metabolic syndrome, overactive bladder, waist circumference

## Abstract

Background

To identify associations between metabolic syndrome (MS) components and overactive bladder (OAB) in women.

Methodology

The present study was conducted prospectively between February 2021 and April 2021 and included the assessment of women admitted to the cardiology outpatient clinic and their female relatives. Records were made of the demographic characteristics of patients and blood tests, including cholesterol, high-density lipoproteins (HDL), low-density lipoproteins (LDL), triglyceride, and fasting glucose levels (FG). In addition, the score on the Overactive Bladder Questionnaire-8-item (OAB-V8) form was noted. The study population was divided into two groups according to OAB-V8 score. The groups were compared in terms of participant demographic properties, OAB-V8 scores, metabolic component values, and blood test results.

Results

In total, 200 participants with a mean age of 49.8 years were enrolled in the study. Participants with OAB had significantly higher body mass index (BMI) (30.1 kg/m^2^ versus 27.1 kg/m^2^; p = 0.001) and longer waist circumference (97.8 cm versus 89.0 cm; p = 0.001). Similarly, the mean FG and LDL levels were significantly higher in participants with OAB (p = 0.001 and p = 0.001). Lastly, mean OAB-V8 score was 20.2 for participants with OAB and 4.8 for participants without OAB. Multivariate regression analysis showed that higher BMI and longer waist circumference were significantly associated with OAB (1.228-fold; p = 0.001 and 1.058-fold; p = 0.001, respectively). Additionally, multivariate regression analysis found that higher LDL level and FG were predictive factors for OAB (1.115-fold; p = 0.003 and 1.229-fold; p = 0.001, respectively).

Conclusions

The present study found that higher BMI, longer waist circumference, and higher LDL and FG levels were predictive factors for OAB development in women.

## Introduction

Overactive bladder (OAB) is characterized by voiding urgency, frequency, and nocturia, with or without urinary incontinence, in the absence of pathologies that can cause these symptoms [1]. Although OAB is not a life-threatening disease, it is associated with deterioration of quality of life, social isolation, and cost to the healthcare system [2]. The true incidence of OAB is still unknown, and previous studies have reported OAB incidence in a wide range [3]. Moreover, the pathophysiology of OAB is not well understood, and morphologic changes in detrusor muscle, neuroplasticity, inflammation, and pelvic ischemia are believed to be among the underlying factors [4]. Recently, many studies have investigated the correlation between OAB and other diseases, including glucose intolerance, hypertension, nervous system diseases, and metabolic syndrome (MS) [5,6].

MS was first described in the late 90s to define cardiovascular risk factors, including dyslipidemia, hypertension, insulin resistance, and obesity. As a result of reduced physical activity and changing dietary habits, MS has become a pandemic disease all around the world [7]. Aguilar et al. reported that one-third of Americans meet the criteria for MS [8]. In another study, Sigit et al. stated that 28% of men and 46% of women were affected by MS in Indonesia [9]. Due to the effects of MS such as glucose intolerance and pelvic ischemia, its relationship with urological diseases is under close observation.

Although previous studies discussed the connection between MS and OAB, there is no consensus on the effect of MS components on OAB. Additionally, studies investigating the relationship between MS components and OAB in women are limited. In the present study, we aimed to identify associations between MS components and OAB in women.

## Materials and methods

The present study was conducted prospectively between February 2021 and April 2021 and included the assessment of women admitted to the cardiology outpatient clinic and their female relatives. All participants signed informed consent forms for enrollment in the study, and the study was planned in accordance with the Helsinki Declaration. To diagnose OAB, all participants completed the Overactive Bladder Questionnaire-8-item (OAB-V8) form (validated Turkish version). Exclusion criteria included the inability to complete the OAB-V8 form, presence of diseases which may be associated with OAB (neurologic disorders, psychiatric illnesses, urinary system malignancies, gynecologic cancers), and being <18 years old. Additionally, patients with active urinary system infection and those with a history of pelvic surgery and pelvic radiation were excluded from the study.

Records were made of the demographic characteristics of patients, including age (age), body mass index (BMI) (kg/m^2^), smoking status, waist circumference (cm), presence of constipation, history of hysterectomy and hypertension, and menopause status. Additionally, blood tests including cholesterol, high-density lipoproteins (HDL), low-density lipoproteins (LDL), triglyceride, and fasting glucose (FG) levels were conducted. In addition, the score on OAB-V8 form was noted.

Metabolic syndrome criteria

The presence of MS is proven if three or more of the following five criteria are met: waist circumference over 35 inches for women, blood pressure higher than 130/85 mmHg, fasting triglyceride level higher than 150 mg/dL, fasting HDL level less than 50 mg/dL for women, and FG level over 100 mg/dL.

Overactive Bladder Questionnaire-8-item

The OAB-V8 form includes eight questions to define the presence and severity of OAB symptoms, including urgency, frequency, nocturia, and urgency urinary incontinence. Patients answer questions on their own and the total OAB-V8 score is calculated by adding the scores for all answers. OAB-V8 scores 8 or higher indicate the presence of OAB and OAB-V8 scores less than 8 indicate the absence of OAB.

The study population was divided into two groups according to the OAB-V8 score. Participants with OAB-V8 score ≥8 were categorized as group 1 and those with OAB-V8 score were <8 classified as group 2. The groups were compared in terms of demographic properties, OAB-V8 scores, metabolic component parameters, and blood test results.

Statistical analysis

Statistical analysis was performed using SPSS version 25 (IBM Corp., Armonk, NY, USA). Normality of distribution of the variables was evaluated using Shapiro-Wilk test and Q-Q plots. The Student’s t-test was chosen for comparison of the normally distributed variables, and non-normally distributed values were evaluated using the Mann-Whitney U test. Quantitative data were expressed as mean ± standard deviation values. Categorical variables were classified and analyzed using the Chi-square test or Fisher’s exact test. Binary logistic regression analysis was chosen to evaluate risk factors for the presence of OAB. The data were analyzed at 95% confidence level, and p values of less than 0.05 were accepted as statistically significant.

## Results

At the end of the evaluation, 200 participants with a mean age of 49.8 ± 9.1 years were enrolled in the study. The mean BMI was 28.0 ± 4.3 kg/m^2^, and 31.5% of the study participants were smokers. The mean HDL, LDL, and triglyceride levels were 50.0 ± 4.4 mg/dL, 115.1 ± 15.5 mg/mL, and 153.3 ± 26.1 mg/dL, respectively. Furthermore, the mean FG level was 103.5 ± 9.5 mg/dL. The mean waist circumference was 91.9 ± 14.5 cm. In total, 69 (34.5%) participants had hypertension and 74 (37%) were post-menopausal. Demographic characteristics and blood test outcomes of participants are summarized in Table 1.

**Table 1:** Demographic data of all patients. *mean ± standard deviation BMI: body mass index; HDL: high-density lipoprotein; LDL: low-density lipoprotein; FG: fasting glucose

	n = 200
Age (years)*	49.8±9.1
BMI (kg/m²)*	28.0±4.3
Smoking status	63 (31.5%)
LDL (mg/mL)*	115.1±15.5
HDL (mg/dL)*	50.0±4.4
Cholesterol (mg/dL)*	205.5±28.7
Triglyceride (mg/dL)*	153.3±26.1
FG (mg/dL)*	103.5±9.5
Waist circumference (cm)*	91.9±14.5
Constipation	37 (17.5%)
Hysterectomy	21 (10.5%)
Hypertension	69 (34.5%)
Post-menopausal status	74 (37.0%)

Comparison of participants with and without OAB revealed that age, smoking status, HDL level, cholesterol level, and triglyceride level were similar between the groups (p = 0.986, p = 0.784, p = 0.766, p = 0.910, and p = 0.424, respectively). Moreover, the presence of constipation, hysterectomy history, hypertension rate, and post-menopausal status were comparable between groups (p = 0.743, p = 0.527, p = 0.980, and p = 0.679, respectively). However, participants with OAB had significantly higher BMI (30.1 kg/m^2^ versus 27.1 kg/m^2^; p = 0.001) and longer waist circumference (97.8 cm versus 89.0 cm; p = 0.001). Similarly, the mean FG and LDL levels were significantly higher in participants with OAB (p = 0.001 and p = 0.007, respectively). Lastly, mean OAB-V8 score was 20.2 for participants with OAB and 4.8 for participants without OAB (p = 0.001) (Table 2).

**Table 2:** Comparison of demographic data of patients between groups. *mean ± standard deviation BMI: body mass index; HDL: high-density lipoprotein; LDL: low-density lipoprotein; FG: fasting glucose; OAB: overactive bladder; OAB-V8: Overactive Bladder Questionnaire-8-item

	OAB (n = 64)	Non-OAB (n = 136)	P-Value
Age (years)	49.8±9.1	49.7±9.2	0.986
BMI (kg/m²)	30.1±3.4	27.1±4.3	0.001
Smoking status	21 (32.8%)	41 (30.1%)	0.784
LDL (mg/mL)	119.5±17.5	113.1±14.2	0.007
HDL (mg/dL)	50.2±4.5	50.6±4.4	0.766
Cholesterol (mg/dL)*	205.1±26.9	205.6±25.9	0.910
Triglyceride (mg/dL)*	155.2±27.9	152.0±25.3	0.424
FG (mg/dL)*	111.7±8.2	99.6±7.3	0.001
Waist circumference (cm)	97.8±15.3	89.0±13.1	0.001
Constipation	11 (17.2%)	26 (19.1%)	0.743
Hysterectomy	8 (12.5%)	13 (9.6%)	0.527
Hypertension	22 (34.3%)	47 (34.6%)	0.980
Post-menopausal status	25 (39.1%)	49 (36.1%)	0.679
OAB-V8 score*	20.2±7.3	4.8±2.1	0.001

Multivariate regression analysis showed that age and presence of hypertension were not risk factors for OAB (p = 0.427 and p = 0.538, respectively). In contrast, higher BMI and longer waist circumference were significantly associated with OAB increment risk (1.228-fold; p = 0.001 and 1.058-fold; p = 0.001, respectively). Additionally, multivariate regression analysis found that higher LDL and FG levels were predictive factors for OAB (1.115-fold; p = 0.003 and 1.229-fold; p = 0.001, respectively) (Table 3).

**Table 3:** Risk factors for overactive bladder risk according to multivariate regression analysis. BMI: body mass index; LDL: low-density lipoprotein; FG: fasting glucose

	Odds ratio	95% Confidence interval	P-Value
Age (years)	1.021	0.970-1.075	0.427
BMI (kg/m²)	1.228	1.091-1.382	0.001
LDL (mg/mL)	1.115	0.986-1.044	0.003
FG (mg/dL)	1.229	1.154-1.310	0.001
Waist circumference (cm)	1.058	1.025-1.091	0.001
Hypertension	1.337	0.530-3.373	0.538

## Discussion

The number of people diagnosed with MS has increased rapidly all over the world in the last few decades. In addition, studies show that MS affects not only the cardiac system but is also related with many diseases, including asthma, cancer, kidney stone, and polycystic ovarian syndrome [10]. In the present study, we focused on the relationship between MS parameters and OAB. We found that longer waist circumference, higher BMI, and higher LDL and FG levels were significantly associated with OAB.

Abnormal glucose metabolism is associated with pathological changes in the bladder, including deterioration in muscle contractility, increased oxidative stress, polyneuropathy, inflammation, and irregularity in urothelial sensitivity [11]. Liu et al. investigated the association between type 2 diabetes mellitus and OAB, and reported that the duration of diabetes mellitus was a predictive factor for OAB, but not HbA1c. Additionally, the authors did not include FG in the study [12]. In another study, Yuksel et al. did not find a significant correlation between FG and OAB, but FG was in the normal range for patients with and without OAB [13]. In the present study, we observed significantly higher FG in participants with OAB. We explain this using the normal glucose levels in the participants with OAB (99.6 mg/dL); however, mean FG was 111.7 mg/dL in participants with OAB, which is considered as pre-diabetes.

Pelvic ischemia caused by atherosclerosis is one of the most accepted hypotheses for OAB development. Azadzoi et al. showed that bladder ischemia is associated with reductions in cellular energy production, deterioration in protein synthesis, and increment in cellular immune cellular events, which play a role in OAB development [14]. In addition, the association between atherosclerosis and high LDL level has been proven. Kilinc et al. investigated the role of LDL in OAB development in the geriatric population, and reported significantly higher LDL levels in patients with OAB using univariate analysis. However, they did not use multivariate regression analysis [15]. In another study, Yuksel et al. stated that LDL level ≥100 m/dL increased OAB risk by 2.8-fold in post-menopausal women [13]. Similarly, we determined that LDL was a predictive factor for OAB syndrome in the present study.

Previous reports investigating the relationship between BMI and OAB reported controversial results. Palma and colleagues interviewed 1,045 women to define correlations between BMI and OAB, and claimed that obese women had significantly higher OAB symptoms than those with normal BMI [16]. In another study, Al-Shaiji and Radomski evaluated the effect of obesity on OAB and urodynamic parameters [17]. In contrast to Palma et al., Al-Shaiji and Radomski found a significant correlation between higher BMI and urinary leakage, but not OAB. Our study suggested that higher BMI was a risk factor for OAB.

Recent studies have emphasized the importance of obesity type and fat distribution as much as obesity; however, studies investigating the effect of waist circumference on OAB are limited and inconsistent. Central obesity is associated with increased pressure on the bladder, and fat tissue acts like a neuroendocrine organ that produces inflammatory factors and induces the sympathetic nervous system [18]. Lai et al. claimed that longer waist circumference was associated with significantly higher prevalence of urge and mixed urinary incontinence in both sexes, as well as higher prevalence of stress urinary incontinence in women [19]. In another study, Elbaset et al. found a significant relationship between waist circumference and OAB on univariate analysis, but multivariate regression analysis found that waist circumference was poorly correlated with OAB risk [20]. In the present study, we found significantly longer waist circumference in participants with OAB.

The present study has some limitations. First, the relatively small participant number is considered a limitation of the study. Second, because the study was cross-sectional in nature, we did not evaluate the period required for OAB development and the influence of relevant parameters. We believe that the interval period between the beginning of OAB and MS could be a subject for another study. Third, the study was conducted in a single academic center. Additionally, we did not investigate the effect of MS on response to treatment for OAB. We believe that the influence of MS on the success of OAB treatment may be a topic for another study.

## Conclusions

The present study found that higher BMI and longer waist circumference were predictive factors for OAB in women. Moreover, higher LDL and FG levels increased the risk of OAB development by 1.115- and 1.229-fold, respectively. Our study suggests that women with MS should be examined for OAB to improve their quality of life and prevent OAB-related complications. Our study findings must be supported by further studies with prospective design and larger patient volume.
